# Membrane-Bound Redox Enzyme Cytochrome *bd*-I Promotes Carbon Monoxide-Resistant *Escherichia coli* Growth and Respiration

**DOI:** 10.3390/ijms25021277

**Published:** 2024-01-20

**Authors:** Martina R. Nastasi, Vitaliy B. Borisov, Elena Forte

**Affiliations:** 1 Department of Biochemical Sciences, Sapienza University of Rome, 00185 Rome, Italy; martinaroberta.nastasi@uniroma1.it; 2 Belozersky Institute of Physico-Chemical Biology, Lomonosov Moscow State University, 119991 Moscow, Russia

**Keywords:** redox enzyme, terminal oxidase, cytochrome, heme, respiratory chain, enzyme inhibition, molecular bioenergetics

## Abstract

The terminal oxidases of bacterial aerobic respiratory chains are redox-active electrogenic enzymes that catalyze the four-electron reduction of O_2_ to 2H_2_O taking out electrons from quinol or cytochrome *c*. Living bacteria often deal with carbon monoxide (CO) which can act as both a signaling molecule and a poison. Bacterial terminal oxidases contain hemes; therefore, they are potential targets for CO. However, our knowledge of this issue is limited and contradictory. Here, we investigated the effect of CO on the cell growth and aerobic respiration of three different *Escherichia coli* mutants, each expressing only one terminal quinol oxidase: cytochrome *bd*-I, cytochrome *bd*-II, or cytochrome *bo*_3_. We found that following the addition of CO to *bd*-I-only cells, a minimal effect on growth was observed, whereas the growth of both *bd*-II-only and *bo*_3_-only strains was severely impaired. Consistently, the degree of resistance of aerobic respiration of *bd*-I-only cells to CO is high, as opposed to high CO sensitivity displayed by *bd*-II-only and *bo*_3_-only cells consuming O_2_. Such a difference between the oxidases in sensitivity to CO was also observed with isolated membranes of the mutants. Accordingly, O_2_ consumption of wild-type cells showed relatively low CO sensitivity under conditions favoring the expression of a *bd*-type oxidase.

## 1. Introduction

Carbon monoxide (CO) is a well-known gaseous molecule that has long been recognized to mediate important physiological processes when produced in low amounts [1,2,3,4,5,6,7,8,9,10,11,12,13,14,15,16,17,18,19,20,21,22]. In eukaryotes, CO is formed endogenously as a byproduct upon the degradation of heme to biliverdin and iron catalyzed by heme oxygenase [23]. In bacteria, this gaseous molecule is generated by homologs of eukaryotic heme oxygenases and via alternative CO-producing mechanisms [24]. Interestingly, CO is considered as a probable signaling molecule between the host and the gut microbiome [24]. Some bacteria can also use CO as a source of energy and carbon [25]. High concentrations of CO are toxic, and some pathogenic bacteria were reported to be susceptible either to CO produced by the host heme oxygenases or to transition-metal-based CO-releasing molecules (CORMs) [26,27]. CORMs developed to deliver physiologically relevant levels of CO experimentally or therapeutically [28,29] showed an additive effect when combined with other antibiotics in certain microbes [26,27]. However, care should be taken as one of the most widely used CORMs, the water-soluble Ru-containing CORM-3, was reported to exert cytotoxic effects due to a thiol-reactive Ru(II) ion and releases little CO [30]. Thus, the development of novel effective CO-releasing drugs is an urgent problem as the therapeutic use of CO has emerged as an antimicrobial strategy in medicine. Bacterial proteins, which contain a pentacoordinate high-spin heme in the reduced state, should bind CO as a strong exogenous ligand, and this, in turn, should affect their function. The terminal oxidases of bacterial respiratory chains have such a heme in their active sites and therefore are among these proteins. These redox-active enzymes belong to class EC 7 translocases. Terminal oxidases catalyze four-electron reduction of O_2_ to 2H_2_O at the expense of oxidation of the respiratory substrate, either quinol or ferrocytochrome *c*. This redox reaction is coupled to the generation of proton-motive force which serves as the driving force for ATP synthesis and other useful work. There are two different groups of these enzymes found in bacteria: heme-copper oxidases, including *aa*_3_-type cytochrome *c* oxidase and *bo*_3_-type quinol oxidase, and copper-lacking *bd*-type quinol oxidases, also called cytochromes *bd* [31,32,33,34,35,36,37,38,39,40,41,42,43,44,45,46,47,48,49,50,51]. Heme-copper oxidases are true proton pumps, whereas cytochromes *bd* generate proton-motive force solely due to the vector transfer of protons along the intraprotein proton-conducting pathway, without a mechanism of proton pumping [47,52,53,54]. *Escherichia coli* is a ubiquitous member of the gut microbiome of humans and warm-blooded animals. Like many other aerobic bacteria, it contains the branched and flexible O_2_-dependent respiratory chain. In the chain, type I and type II NADH dehydrogenases transfer electrons from NADH to ubiquinone-8 and/or menaquinone-8. Ubiquinone-8 can also accept electrons from succinate via succinate dehydrogenase. Then, electrons from ubiquinol-8 and/or menaquinol-8 are transferred by three terminal oxidases, cytochromes *bo*_3_, *bd*-I, and *bd*-II, to the final electron acceptor, O_2_, to produce 2H_2_O [55,56,57,58,59].

In *E*. *coli*, the *bo*_3_, *bd*-I, and *bd*-II oxidases are encoded by the *cyoABCDE*, *cydABX*, and *appCBX* operons, respectively. With the use of a rigorous chemostat methodology and powerful modeling tools, it was shown that the *cyoABCDE* operon was maximally induced under fully aerobic conditions [60]. The *cydABX* operon was maximally expressed at 56% aerobiosis [61]. The *appCBX* operon was maximally expressed at 0% aerobiosis. Spectroscopic assays of oxidase levels confirmed these conclusions [60,62]. Thus, at high aeration, cytochrome *bo*_3_ is preferentially expressed, whereas a shift from aerobic to microaerobic conditions activates the expression of the *bd*-type cytochromes [55]. The atomic structures of the three cytochromes were reported [43,44,63,64,65,66,67]. Cytochrome *bo*_3_, composed of four subunits, carries the ubiquinol-binding site and three metal redox groups, a low-spin heme *b*, a high-spin heme *o*_3_, and Cu_B_. Heme *b* accepts electrons from ubiquinol; heme *o*_3_ and Cu_B_ compose a binuclear site for O_2_ reduction [55]. Cytochromes *bd*-I and *bd*-II consist of four and three subunits, respectively. Both *bd* enzymes contain the ubiquinol/menaquinol-binding site (called the Q-loop) and three hemes, a low-spin *b*_558_, a high-spin *b*_595_, and a high-spin *d*, but no copper ion. Heme *b*_558_ is the immediate electron acceptor for ubiquinol and/or menaquinol. Heme *d* serves as the O_2_-reducing site. The functional role of heme *b*_595_ is not clear yet, but at least apparently it transfers electrons from heme *b*_558_ to heme *d* [55].

It is worth mentioning that cytochrome *c* and chlorophyll *a* were reported to be suitable redox mediators for the development of enzymatic biofuel cell systems [68]. In these systems, electron transfer from glucose oxidase to the electrode was facilitated through cytochrome *c* and chlorophyll *a* adsorbed on the electrode [68]. In this regard, the applicability of cytochrome *d* and cytochrome *o*_3_ for charge transfer from/to bacteria and in biofuel cells could also be evaluated.

A protective role of cytochrome *bd*-I against nitric oxide [69,70,71], peroxynitrite [72], and ammonia [73] was reported. Cytochrome *bd*-I likely provides NO resistance in *E. coli* for two reasons. The first reason is the extraordinarily high rate of NO dissociation from heme *d*^2+^: the *k*_off_ values were reported to be 0.133 s^−1^ [69] and 0.163 s^−1^ [70]. This is about 30 times higher than that for NO dissociation from heme *a*_3_^2+^ in the mitochondrial cytochrome *c* oxidase [74] and can explain why following inhibition by NO the oxygen reductase activity of the *bd*-I enzyme is restored much faster than that of the mitochondrial enzyme. The second plausible reason is the ability of a *bd* oxidase to rapidly convert NO into NO_2_^–^ in turnover, although that was reported only for cytochrome *bd* from *Azotobacter vinelandii* so far [75]. The fact that the *bd*-I oxidase is not inhibited by peroxynitrite can be due to its ability to catalytically scavenge peroxynitrite [72]. The reaction likely occurs on the heme *d* active site, and at least four possible reaction mechanisms have been suggested [76]. Cytochrome *bd*-I is not only resistant to but also activated by ammonia under alkaline conditions [73]. In this case, ammonia is suggested to react with a few catalytic intermediates of the enzyme. In particular, NH_3_ can promote the formation of the peroxy state (**P**) from the oxidized state (**O**). NH_3_ also possibly reacts with the one-electron-reduced state (**O^1^**) to produce the ferryl state (**F**). In these reactions, NH_3_ presumably serves as a two-electron donor being oxidized to NH_2_OH [73]. 

Both *bd*-type oxidases also contribute to *E. coli* resistance to cyanide [77], hydrogen peroxide [78,79,80], and sulfide [77,81]. On the contrary, the activity of the *bo*_3_ oxidase was shown to be highly sensitive to inhibition by cyanide, sulfide, nitric oxide, and ammonia [70,73,77,81]. Thus, a *bd*-type terminal oxidase endows *E. coli* and possibly other bacteria with resistance to the above-mentioned stressors and, being absent in eukaryotic cells, can serve as a good therapeutic target [82].

Increased expression of cytochrome *bd* is in fact a likely mechanism for survival used by the pathogenic microorganisms in the presence of reactive oxygen and nitrogen species generated by the host immune system to fight infection [59]. In view of the fact that cytochrome *bd*-I exhibits tolerance to such reactive species, it is relevant to examine its potential resistance also to CO, which has been reported to be important in host–pathogen relationships [83,84,85,86] and is a heme ligand like NO. It has to be noted that data on the effect of CO on the function of bacterial terminal oxidases are limited and contradictory. Of the three *E. coli* oxidases, cytochrome *bo*_3_ was shown to be the least sensitive to inhibition by CO if the enzymes were purified and detergent-solubilized [87]. In contrast, according to a recent short report [88], cytochrome *bd*-I is more resistant to inhibition by CO than cytochrome *bd*-II and cytochrome *bo*_3_ if CO is added to *E. coli* cell suspensions, at [O_2_] = 150 μM. Bayly et al. also studied the physiological response of *Mycobacterium smegmatis* to CO [83]. The respiratory chain of mycobacteria is known to contain two different terminal oxidases: cytochrome *bcc*-*aa*_3_ supercomplex and cytochrome *bd* [89]. Bayly et al. reported that in *M. smegmatis* cell cultures, the activity of cytochrome *bd* is resistant to CO while cytochrome *bcc*-*aa*_3_ supercomplex is strongly inhibited by CO [83]. Furthermore, *M. smegmatis* lacking the *bd* oxidase shows a significant growth defect in the presence of this gas [83].

In this work, we studied the effect of CO on aerobic respiration sustained by *bo*_3_, *bd*-I, or *bd*-II oxidases in cell cultures of *E. coli* respiratory mutants by varying the O_2_ concentration at which CO was added, and we examined the ability of these cytochromes to sustain bacterial growth in the presence of CO. We also tested the CO inhibition of O_2_ consumption of both isolated membranes of the mutants and wild-type cells grown under conditions favoring the expression of either cytochrome *bo*_3_ or a *bd*-type oxidase.

## 2. Results 

### 2.1. Effect of CO on E. coli Aerobic Respiration

**Figure 1:**
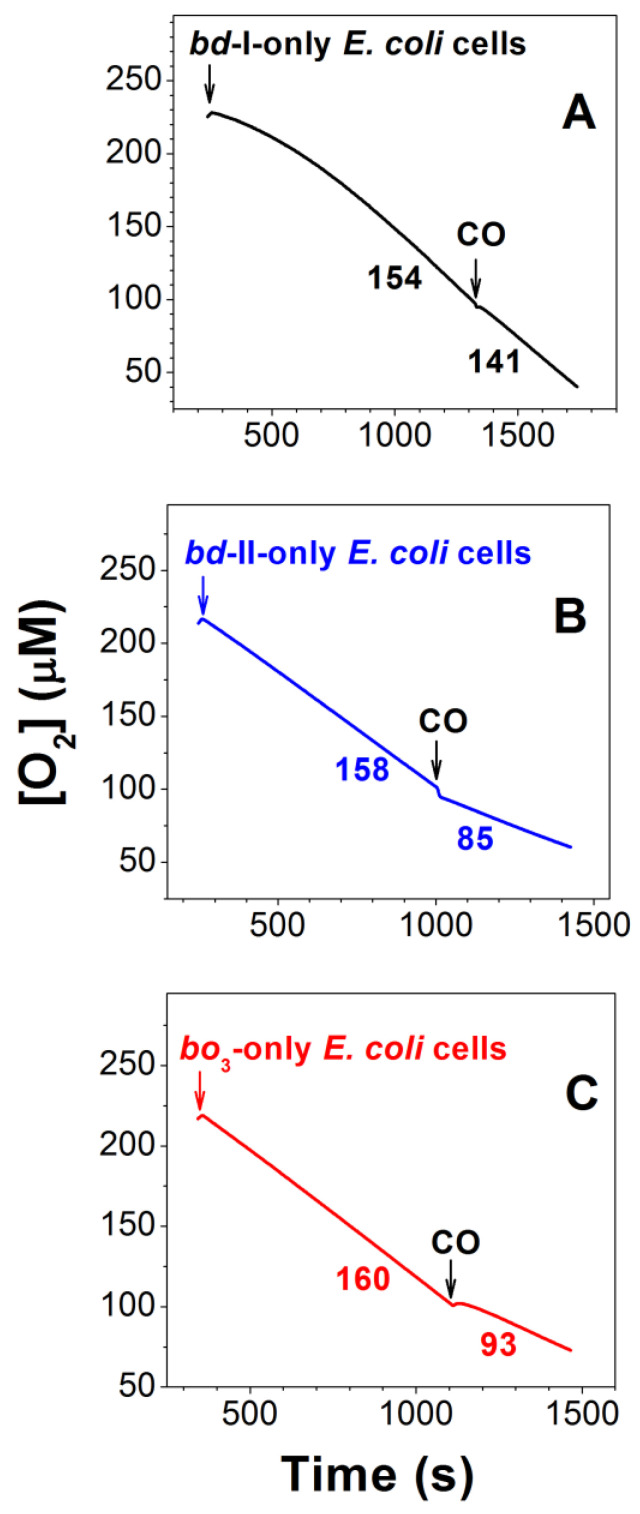
Typical O_2_ consumption traces showing the effect of CO on *E. coli* aerobic respiration in cell suspensions of mutant strains expressing cytochrome *bd*-I (**A**), cytochrome *bd*-II (**B**), or cytochrome *bo*_3_ (**C**) as the sole terminal oxidase. Here, 96.3 μM CO was added at [O_2_] = 100 μM. The O_2_ consumption rates (nM O_2_/s) measured prior to and following the addition of CO are shown adjacent to each trace. Concentrations of *bd*-I, *bd*-II, and *bo*_3_ oxidases were 75, 52, and 47 nM, respectively.

We examined the effect of CO on the aerobic respiration of *E. coli* cells of the three different mutant strains. The aerobic respiratory chain of each mutant contains only one terminal quinol oxidase: cytochrome *bd*-I, cytochrome *bd*-II, or cytochrome *bo*_3_. In each mutant, aerobic cellular respiration was supplied by endogenous electron-donor respiratory substrates; therefore, the use of an exogenous reducing system was not required. Figure 1A shows that 96.3 μM CO added at [O_2_] = 100 μM inhibits O_2_ consumption by *bd*-I-only *E. coli* cells to a small extent of 11.6 ± 1.1%. Under these experimental conditions, the CO inhibitory effect on the aerobic respiration of *bd*-II-only and *bo*_3_-only *E. coli* cells is much greater, 43.3 ± 7.6% and 44.3 ± 1.5%, respectively (Figure 1B,C).

**Figure 2:**
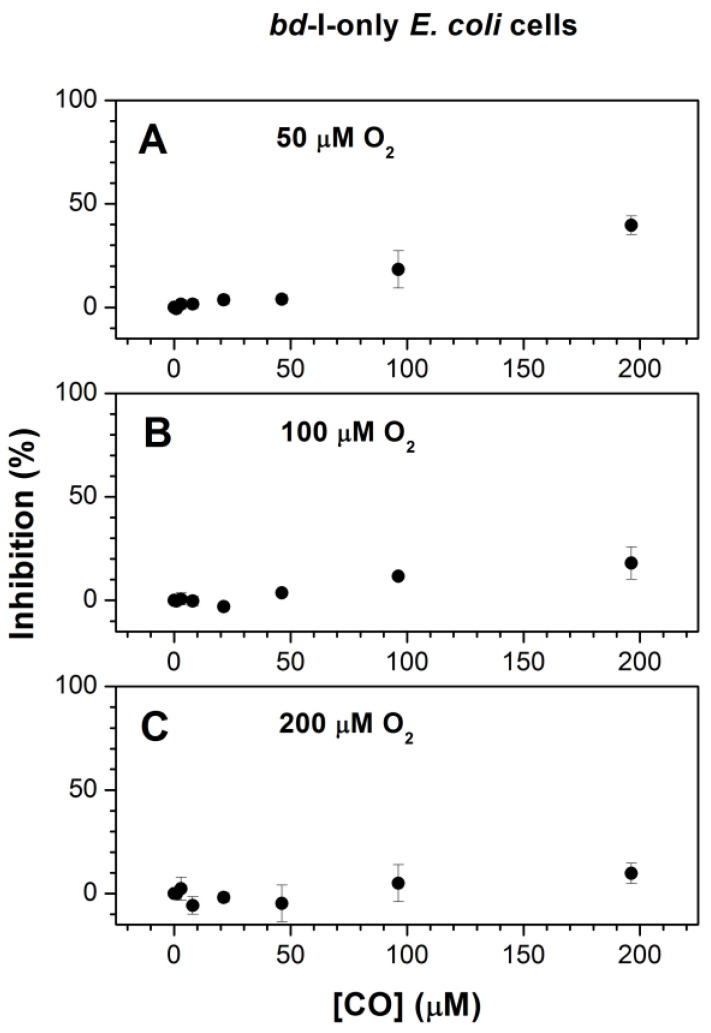
CO inhibition of aerobic respiration of cell suspensions of *E. coli* mutant strain expressing cytochrome *bd*-I as the sole terminal oxidase at different O_2_ concentrations. Measurements were performed at increasing [CO] added at [O_2_] = 50 μM (**A**), 100 μM (**B**), and 200 μM (**C**). Concentrations of *bd*-I oxidase in experiments in which CO was added at 50, 100, and 200 μM O_2_ were 66 ± 15, 64 ± 9, and 65 ± 10 nM, respectively. Values represent the mean (*n* = 3) ± standard deviation.

**Figure 3:**
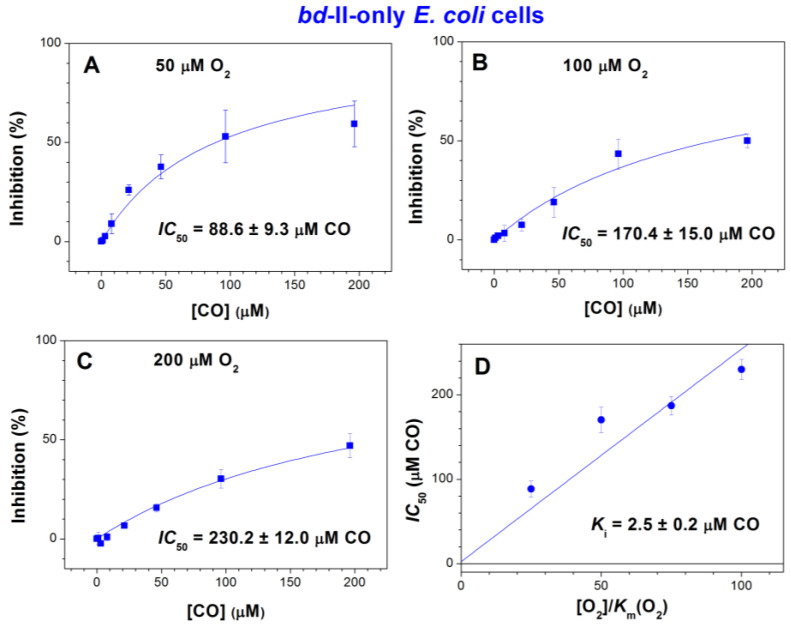
CO inhibition of aerobic respiration of cell suspensions of *E. coli* mutant strain expressing cytochrome *bd*-II as the sole terminal oxidase at different O_2_ concentrations. (**A**–**C**) Determination of apparent *IC*_50_. Square symbols are experimental data points. Measurements were performed at increasing [CO] added at [O_2_] = 50 μM (**A**), 100 μM (**B**), and 200 μM (**C**). (**D**) Determination of *K*_i_. It was estimated using the depicted *IC*_50_ values (circle symbols), assuming competitive inhibition. The *IC*_50_ value obtained at [O_2_] = 150 μM (187.1 ± 11.1 μM CO) was taken from [88]. The apparent *K*_m_(O_2_) value (2 µM) was taken from [90]. Concentrations of *bd*-II oxidase in experiments in which CO was added at 50, 100, and 200 μM O_2_ were 53 ± 11, 51 ± 8, and 51 ± 11 nM, respectively. Values represent the mean (*n* = 3) ± standard deviation.

**Figure 4:**
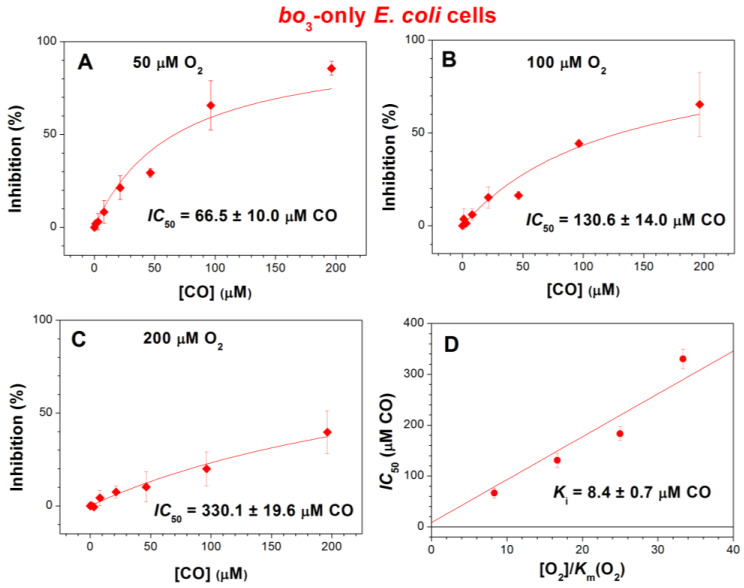
CO inhibition of aerobic respiration of cell suspensions of *E. coli* mutant strain expressing cytochrome *bo*_3_ as the sole terminal oxidase at different O_2_ concentrations. (**A**–**C**) Determination of apparent *IC*_50_. Rhombus symbols are experimental data points. Measurements were performed at increasing [CO] added at [O_2_] = 50 μM (**A**), 100 μM (**B**), and 200 μM (**C**). (**D**) Determination of *K*_i_. It was estimated using the depicted *IC*_50_ values (circle symbols), assuming competitive inhibition. The *IC*_50_ value obtained at [O_2_] = 150 μM (183.3 ± 13.5 μM CO) was taken from [52]. The apparent *K*_m_(O_2_) value (6 µM) was taken from [70]. Concentrations of *bo*_3_ oxidase in experiments in which CO was added at 50, 100, and 200 μM O_2_ were 58 ± 17, 56 ± 9, and 57 ± 9 nM, respectively. Values represent the mean (*n* = 3) ± standard deviation.

We compared the inhibition of respiration of the three mutants by increasing [CO] added at three different O_2_ concentrations, 50, 100, and 200 μM (Figure 2, Figure 3 and Figure 4). In the case of *bd*-I-only cells, at [O_2_] = 50, 100, and 200 μM, the maximum inhibition percentage at a maximum concentration of added CO, 196.3 μM, appeared to be 39.6 ± 4.5%, 18.0 ± 7.8%, and 9.7 ± 4.9%, respectively (Figure 2). The respective values were 59.3 ± 11.5%, 50.0 ± 3.6%, and 47.0 ± 6.0% for *bd*-II-only cells (Figure 3A–C) and 85.6 ± 3.7%, 65.3 ± 17.2%, and 39.7 ± 11.5% for *bo*_3_-only cells (Figure 4A–C). Thus, at all O_2_ concentrations studied, in *E. coli* cell suspensions, cytochrome *bd*-I turned out to be much more resistant to inhibition by CO than cytochrome *bd*-II or cytochrome *bo*_3_. One can see that in each mutant, the degree of inhibition decreases with increasing [O_2_]. This suggests competitive enzyme inhibition; i.e., in all three terminal oxidases, CO competes with the substrate, O_2_, for binding to the enzyme’s active site under turnover conditions.

As the inhibition of respiration of *bd*-II-only and *bo*_3_-only *E. coli* mutants by CO was significant, we were able to obtain the apparent half-maximal inhibitory concentration values, *IC*_50_, for CO added at different O_2_ concentrations. At [O_2_] = 50, 100, and 200 μM, the respective *IC*_50_ values were 88.6 ± 9.3, 170.4 ± 15.0, and 230.2 ± 12.0 μM CO for *bd*-II-only cells (Figure 3A–C) and 66.5 ± 10.0, 130.6 ± 14.0, and 330.1 ± 19.6 μM CO for *bo*_3_-only cells (Figure 4A–C). In view of the insignificant inhibition, it was not possible to obtain *IC*_50_ for *bd*-I-only cells.

With the *IC*_50_ values at different O_2_ concentrations, we were in a position to estimate the inhibition constants (*K*_i_) for CO in the case of *bd*-II-only and *bo*_3_-only *E. coli* cells. To achieve this goal, the *IC*_50_ values acquired at different [O_2_] values were plotted as a function of [O_2_]/*K*_m_(O_2_). The data were fitted to the appropriate equation assuming a competitive mode of inhibition [91]. For analysis purposes, the previously reported *K*_m_(O_2_) values, 2 µM (for cytochrome *bd*-II [90]) and 6 µM (for cytochrome *bo*_3_ [70]), were used. As a result of this analysis, we obtained the following *K*_i_ values for CO: 2.5 ± 0.2 µM for *bd*-II-only cells (Figure 3D) and 8.4 ± 0.7 µM for *bo*_3_-only cells (Figure 4D).

**Figure 5:**
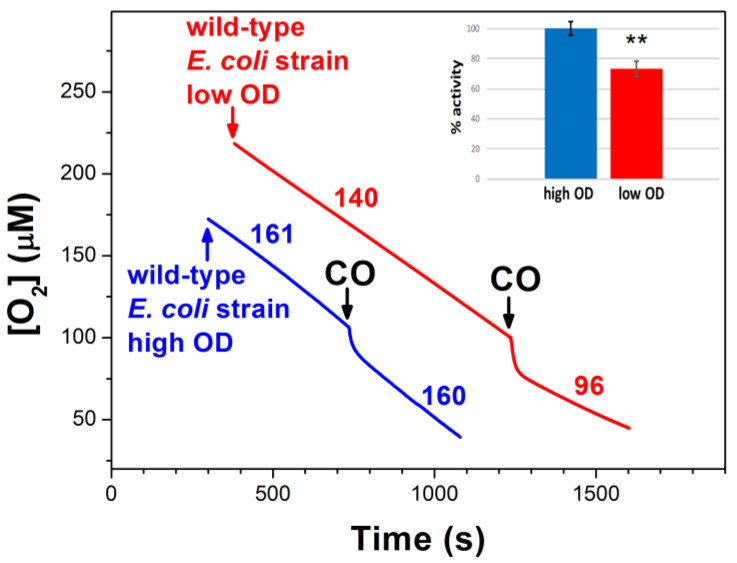
Traces of O_2_ consumption of cell suspensions showing the effect of CO on aerobic respiration of the *E. coli* wild-type (wt) strain MG1655 at high OD (blue line) and low OD (red line), indicating the prevalent expression of the *bd* oxidases and cytochrome *bo*_3_, respectively. Here, 96.3 μM CO was added at [O_2_] = 100 μM. The O_2_ consumption rates (nM O_2_/s) measured prior to and following the addition of CO are shown adjacent to each trace. Additions: 1 mL of cells at 0.48 OD (red line), 0.2 mL of cells at 3 OD (blue line). Inset: Percent activity after CO addition to respiring wild-type cells. Asterisks denote statistically significant differences between wild-type cells grown at high and low OD (**, *p* < 0,01; *t*-test).

We also investigated the effect of CO on the aerobic respiration of wild-type *E. coli* cells. Transcriptomic studies on wild-type *E. coli* cultured at different O_2_ availabilities, coupled with biochemical determination of respiratory oxidase expression [60,61], showed that both the transcript abundance of *cyoA* and *cydA* and the expression of the corresponding *bo*_3_ and *bd* cytochromes are observed under fully aerobic and microaerobic conditions, respectively. Consistently, a change in oxidase expression from cytochrome *bo*_3_ to the *bd*-type cytochromes was reported to occur with cell growth, following a progressive reduction in O_2_ availability in the medium [77]. In agreement with Forte et al. [77], when studying cells in an early growth phase of the culture (OD_600_ < 0.8), most of the respiration (50–70%) is sensitive to 50 μM sulfide, pointing to a prevalent expression of cytochrome *bo*_3_. Conversely, in a late growth phase of the culture (OD_600_ > 2.5), when O_2_ is limiting, sulfide has little effect on respiration, indicating a prevalent expression of the *bd*-type cytochromes. Accordingly, O_2_ consumption of wild-type cells that were harvested at high OD_600_ (the prevalent expression of a *bd*-type oxidase) and low OD_600_ (the prevalent expression of cytochrome *bo*_3_) showed low and high sensitivity to CO, respectively (Figure 5).

**Figure 6:**
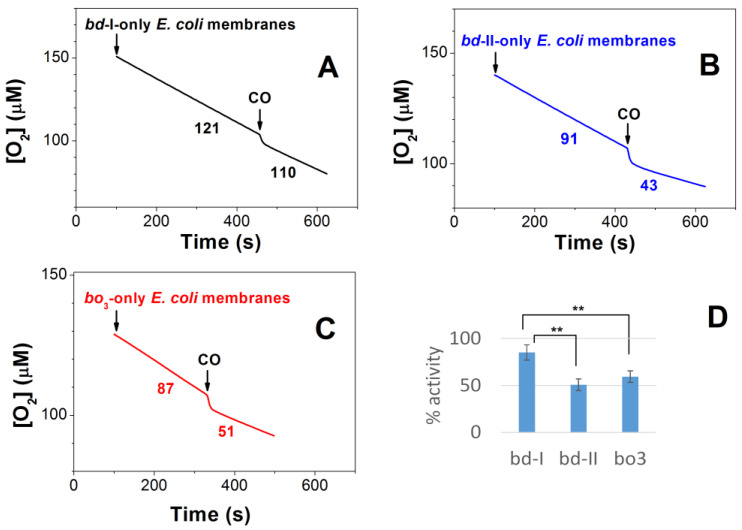
Traces of O_2_ consumption showing the effect of CO on aerobic respiration of isolated membranes from *E. coli* mutant strains expressing cytochrome *bd*-I (**A**), cytochrome *bd*-II (**B**), or cytochrome *bo*_3_ (**C**) as the sole terminal oxidase. (**D**) Percent of O_2_ reductase activity after CO additions to respiring membranes. Asterisks denote statistically significant differences with respect to *bd*-I activity (**, *p* < 0,01; *t*-test). Here, 96.3 μM CO was added at [O_2_] = 100 μM. The O_2_ consumption rates (nM O_2_/s) measured prior to and following the addition of CO are shown adjacent to each trace. Additions: 0.3 mg/mL of *bd*-I-containing isolated membranes (black line), 0.5 mg/mL of *bd*-II-containing isolated membranes (blue line), 0.6 mg/mL of *bo*_3_-containing isolated membranes (red line).

In addition, we tested the CO inhibition of O_2_ consumption of membranes isolated from mutants. As with cell cultures, CO effectively inhibits the O_2_ reductase activity of both *bd*-II- and *bo*_3_-containing membranes, whereas the activity of *bd*-I-containing membranes is relatively resistant to inhibition by CO (Figure 6).

The addition of N_2_ to respiring cells and membranes did not significantly alter the O_2_ consumption rate, indicating that the observed inhibitory effect on wild-type cells at low OD values as well as on *bd*-II and *bo*_3_ mutant strains is due to CO (Supplementary Figures S1 and S2).

### 2.2. Effect of CO on E. coli Cell Growth

**Figure 7:**
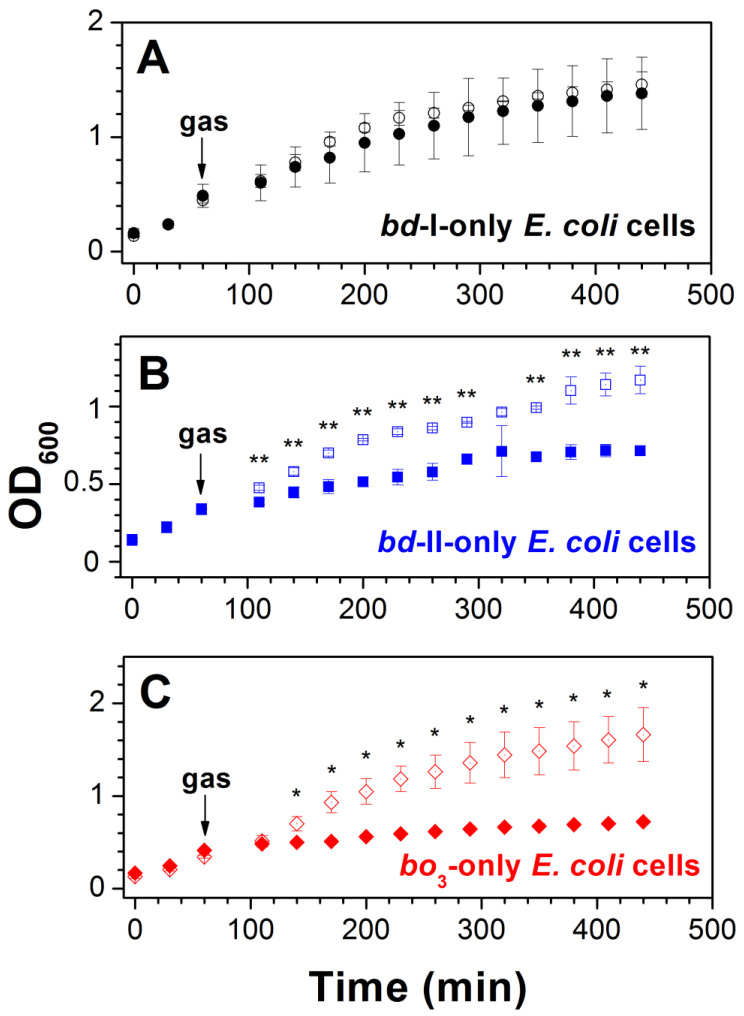
Effect of CO on *E. coli* cell growth. Cell growth of *E. coli* mutant strains expressing cytochrome *bd*-I (**A**), cytochrome *bd*-II (**B**), or cytochrome *bo*_3_ (**C**) as the sole terminal oxidase assayed in the presence of either ~20% CO (‘closed symbols’) or ~20% N_2_ (‘open symbols’). The arrow shows the time (60 min) at which cells were subjected to the gas flushing treatment for 30 s. Values represent the mean (*n* = 3) ± standard deviation. Asterisks denote statistically significant differences between CO- and N_2_-treated cells (*, *p* < 0,05; **, *p* < 0,01; *t*-test).

The observation that the degree of resistance of the O_2_ consumption process by *bd*-I-only *E. coli* cells to CO is quite high, as opposed to the high sensitivity for the gas displayed by *bd*-II-only and *bo*_3_-only cells, prompted us to examine whether cytochrome *bd*-I, beyond enabling aerobic respiration, promotes *E. coli* cell growth in the presence of CO. In order to determine the effect of CO on *E. coli* cell growth, we studied the growth of the three different respiratory mutant strains in the presence of either ~20% CO or ~20% N_2_ as a control. Following the addition of ~20% CO to the *E. coli* strain expressing cytochrome *bd*-I as the only terminal oxidase at 60 min after the start of growth in air, a minimal effect on cell growth was observed (Figure 7A). On the contrary, the growth of both *bd*-II-only and *bo*_3_-only strains was severely impaired over the same time window after the addition of ~20% CO, compared to the control with ~20% N_2_ (Figure 7B,C). Thus, these data suggest that, unlike cytochromes *bd*-II and *bo*_3_, the *bd*-I oxidase promotes *E. coli* growth in the presence of CO.

## 3. Discussion

We examined the effect of CO on the O_2_-dependent respiration of the *E. coli* respiratory mutants containing one of the three terminal oxidases. The experiments were performed at different concentrations of the enzyme substrate, O_2_. In all mutants tested, the inhibitory action of CO decreased with increasing [O_2_]. The fact that the degree of inhibition decreases as the substrate concentration increases clearly suggests that CO acts as a competitive inhibitor for the enzyme-catalyzed O_2_ reduction reaction under steady-state conditions. CO apparently competes with O_2_ for binding to a high-spin pentacoordinate ferrous heme in the enzyme’s active site. Previous spectroscopic studies showed that the redox-active group that, in the reduced state, is able to bind both O_2_ and CO is heme *d* in the *bd*-type oxidases and heme *o*_3_ in the *bo*_3_ oxidase [87,92,93]. For this reason, we think that in the present enzyme-inhibition experiments with *E. coli* cells, heme *d* and heme *o*_3_ are targets for CO in cytochromes *bd*-I/*bd*-II and cytochrome *bo*_3_, respectively.

We found that the *bd*-I oxidase is much less sensitive to CO than both the *bd*-II and *bo*_3_ oxidases, as confirmed by measuring the effect of CO on O_2_ consumption by both whole cells and isolated membranes of the respiratory mutants. Accordingly, the O_2_ consumption of wild-type cells displayed low CO sensitivity under conditions favoring the expression of a *bd*-type oxidase and high CO sensitivity when cytochrome *bo*_3_ was predominantly expressed. Consistently, cell growth proved to be almost unaffected by CO in an *E. coli* mutant strain expressing solely cytochrome *bd*-I, but was drastically inhibited after the addition of the gas to mutant strains expressing either cytochrome *bo*_3_ or cytochrome *bd*-II as the only terminal oxidase. These results are in disagreement with an earlier study according to which O_2_ consumption by the isolated cytochromes *bd*-I and *bd*-II is more sensitive to inhibition by CO than that by the isolated *bo*_3_ oxidase [87]. The measurement conditions, such as buffer, pH, and temperature, in [87] and in this work were the same, but the difference was the environment for the enzyme (detergent in [87] versus natural lipid bilayer in this study). Therefore, we assume that this inconsistency can be explained by differences in the protein environment which affect enzyme sensitivity towards CO. In this work, the oxidases were in vivo conditions, i.e., integrated into native bacterial membranes, whereas in [87], the enzymes were isolated and incorporated into detergent micelles. Indeed, more and more data are accumulating on how the lipid membrane environment can significantly affect the structure, function, and dynamics of various membrane proteins [94,95]. For instance, regarding terminal oxidases, it was shown that the membrane environment modulates the ligand-binding characteristics of the *E. coli* cytochrome *bd*-I [96]. According to another study, solubilization of the membrane-bound bovine cytochrome *c* oxidase leads to an increase in the binding affinity of the enzyme for cyanide by 100–1000 times [97]. In addition, it was reported that in *E. coli* membranes, the *bd*-I oxidase is apparently in a supercomplex with other membrane-bound respiratory enzymes [56]. Such protein–protein interactions could also modulate the sensitivity of cytochrome *bd*-I to CO.

We hypothesize that when the *E. coli* cytochromes are integrated into the native lipid bilayer, the binding affinities of the enzymes for CO and O_2_ are such that CO binding to heme *d* in the *bd*-I oxidase is outcompeted by O_2_ while the binding of the inhibitor to heme *d* in the *bd*-II oxidase or to heme *o*_3_ in the *bo*_3_ oxidase is not. The resistance of cytochrome *bd*-I to CO is in agreement with the extremely fast dissociation of CO from the oxidase (*k*_off_ = 6.0 ± 0.2 s^−1^ for the fully reduced form of the CO-bound enzyme [69]). Such a high enzyme–ligand dissociation rate constant would indeed lead to the prompt restoration of respiration. The variation between the terminal oxidases in the affinity for the ligands may arise from differences in the structural organization of the O_2_-binding sites and their specific environment. Consistently, among the factors that can regulate the binding affinity of heme proteins for gaseous ligands are the chemical structure and geometry of the proximal axial ligand of the heme and its distal amino acid residues [98]. Distal amino acid residues may either stabilize the bound diatomic gaseous molecule via weak interactions, including electrostatic effects, hydrogen bonds, van der Waals interactions, and the hydrophobic effect, or, contrariwise, destabilize ligand binding by providing steric constraints [98,99].

It is of interest to mention global *E. coli* responses to CO. Transcription factor measurements and modeling showed that gene expression is significantly perturbed by CO, with major consequences for energy metabolism, iron homeostasis, and amino acid metabolism [86]. Genes encoding energy-transducing proteins are highly affected by CO via the global regulators, ArcA and FNR. CO inhibition of respiration results in over-reduction of the quinone pool; accumulation of the fermentation product, pyruvate; and enhanced expression of iron acquisition genes. Regarding the respiratory terminal oxidases, the transcriptomic analysis of wild-type *E. coli* exposed to CO gas revealed that under aerobic conditions there is a 5- to 10-fold decrease in the expression of the *cyoABCDE* operon, encoding cytochrome *bo*_3_. In contrast, there is a 4-fold increase in the expression of the *cydABX* operon, encoding cytochrome *bd*-I. Changes in the expression of the *appCBX* operon, encoding cytochrome *bd*-II oxidase, are slight [86]. The CO inhibition data reported here are consistent with the transcriptional response to CO in wild-type cells reported by Wareham et al. [86]. Indeed, it seems reasonable that following CO gas addition, the CO-sensitive *bo*_3_ oxidase is downregulated and the CO-*in*sensitive *bd*-I oxidase is upregulated. The *bd*-II oxidase normally is not expressed under aerobic conditions, and there is no need for its upregulation after CO treatment since the enzyme is sensitive to CO. 

The difference in susceptibility to CO inhibition between cytochromes *bd*-I and *bo*_3_ is indeed not unexpected since they belong to different superfamilies of terminal oxidases and, as reported before, also differ in resistance to other stressors, such as cyanide, sulfide, nitric oxide, and ammonia [70,73,77,81]. However, the fact that cytochrome *bd*-I is much more resistant to CO than cytochrome *bd*-II in respiring *E. coli* cells is surprising. In order to explain this phenomenon, the following structural differences between the two *bd* enzymes have to be noted. First, the two proteins differ in the number of subunits. The *bd*-I oxidase is composed of four subunits, namely CydA, CydB, CydX, and CydY, whereas the *bd*-II oxidase contains one fewer subunit, being composed of AppC, AppB, and AppX. Notably, the extra subunit in cytochrome *bd*-I, CydY, shields the high-spin pentacoordinate heme *b*_595_ from the lipid bilayer interface. This shielding prevents external ligands from accessing this potential ligand-binding site [64,65]. Consistently, heme *b*_595_ resistance to external ligand binding in cytochrome *bd*-I was previously identified in an MCD study [92]. Since cytochrome *bd*-II has no CydY, direct access of gaseous molecules from the membrane lipid environment is possible [66,67]. Therefore, in the *bd*-II oxidase, CO could possibly bind to the reduced heme *b*_595_ under turnover conditions. This binding would, in turn, inhibit the electron transfer from heme *b*_558_ to heme *d* that occurs through heme *b*_595_, leading to the inhibition of the enzyme-catalyzed O_2_ consumption. Second, it was reported that in the *bd*-II oxidase, the axial heme *d* ligand is His^19^ of the AppC subunit [66,67]. However, there is no consensus on the nature of the axial ligand of heme *d* in the *bd*-I oxidase. It is either His^19^ (of the CydA subunit, homolog to AppC) [64] or Glu^99^ of CydA [65]. Notably, His^19^ and Glu^99^ are located on opposite sides of the plane of the porphyrin macrocycle. If the axial heme *d* ligand in the *bd*-I and *bd*-II enzymes in bacterial cells is indeed different, this may also underlie the difference in sensitivity of the two proteins to CO. The fact that heme *d* in the *bd*-II oxidase has a much higher midpoint redox potential than that in the *bd*-I oxidase (+440 vs. +258 mV) [28]) would be consistent with the different nature of its axial ligand. Third, the *bd*-II protein incorporated into amphipols was shown to be mainly in the form of a dimer, while the *bd*-I enzyme exists only as a monomer [66]. This difference might also contribute to the observed difference in the sensitivity of these two *bd* oxidases to CO. In this regard, it is also worth noting that *Cupriavidus necator* H16, like *E. coli* and some other bacteria, has two different operons encoding cytochrome *bd*, *cydA1B1* and *cydA2B2*. Interestingly, the expression of only one of the two (*cydA2B2*) seems to enable cell growth in the presence of CO under heterotrophic conditions [100]. The deletion of *cydA2B2* had a detrimental effect on CO resistance, and plasmid-based expression of *cydA1B1* did not improve CO tolerance [64]. These data are consistent with our observation that, of the two *bd* oxidases in *E. coli* cells, only cytochrome *bd*-I promotes aerobic respiration and growth in the presence of CO.

**Figure 8:**
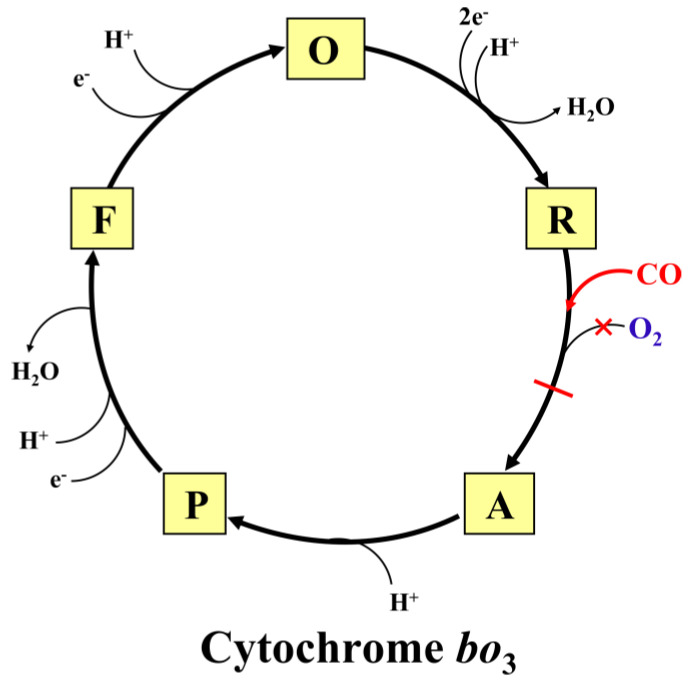
Proposed mechanism for the inhibitory effects of CO on the catalytic cycle of cytochrome *bo*_3_. Catalytic intermediates **O** (*o*_3_^3+^–OH Cu_B_^2+^), **R** (*o*_3_^2+^ Cu_B_^+^), **A** (*o*_3_^2+^–O_2_ Cu_B_^+^), **P** (*o*_3_^4+^=O^2−^ Cu_B_^2+^–OH), and **F** (*o*_3_^4+^=O^2−^ Cu_B_^2+^) are shown. Only chemical protons are shown. Pumped protons are not shown for clarity. The two ferryl intermediates **P** and **F** probably differ in the presence of an aromatic amino acid radical in **P**, as in the **P_M_** species of *aa*_3_-type cytochrome *c* oxidase [101]. CO binds to heme *o*_3_^2+^ in the **R** species, forming the *o*_3_^2+^–CO complex that prevents the binding of O_2_ to heme *o*_3_^2+^, and, as a consequence, leads to the inhibition of the enzyme activity.

**Figure 9:**
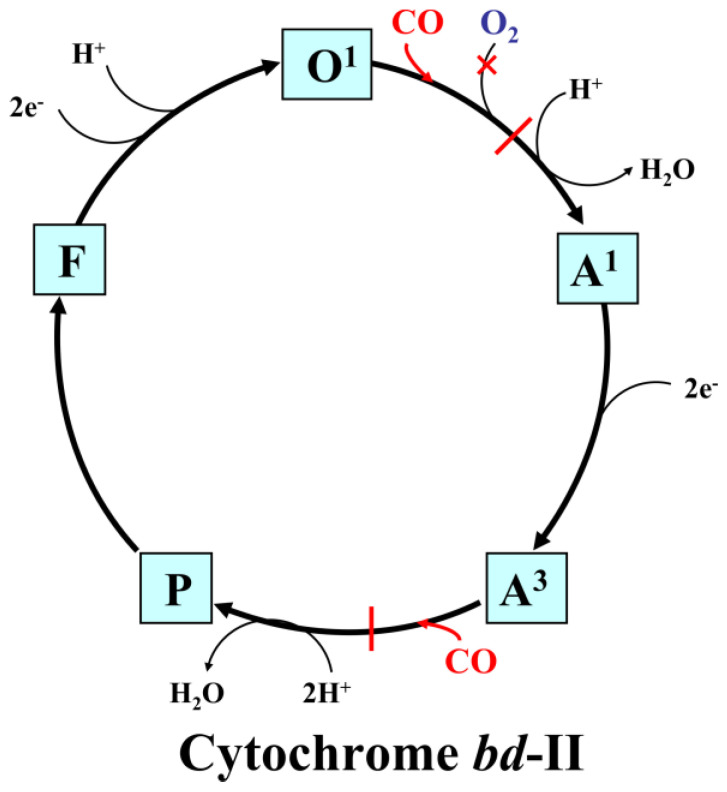
Proposed mechanism for the inhibitory effects of CO on the catalytic cycle of cytochrome *bd*-II. Proposed catalytic intermediates **O^1^** (*b*_558_^2+^ *b*_595_^3+^ *d*^3+^–OH), **A^1^** (*b*_558_^3+^ *b*_595_^3+^ *d*^2+^–O_2_), **A^3^** (*b*_558_^2+^ *b*_595_^2+^ *d*^2+^–O_2_), **P** (*b*_558_^2+^ *b*_595_^3+^ d*^4+^=O^2^ where d*^4+^=O^2^ is a ferryl porphyrin π-cation radical [102,103]), and **F** (*b*_558_^3+^ *b*_595_^3+^ *d*^4+^=O^2−^) are shown. In the **O^1^** species, an electron is probably distributed between the three hemes. The reaction of CO with **O^1^** stabilizes the electron on heme *d*, and the *d*^2+^–CO complex is generated. This, in turn, prevents the binding of O_2_ to heme *d*^2+^ and, as a consequence, leads to the inhibition of the enzyme activity. Furthermore, CO could also bind to heme *b*_595_^2+^ in the **A^3^** species, producing the *b*_595_^2+^–CO complex. This would stabilize heme *b*_595_ in the reduced state and would not allow heme *b*_595_^2+^ to rapidly donate an electron to O_2_ bound to heme *d*^2+^ to perform concerted four-electron reduction of O_2_ to 2H_2_O.

**Figure 10:**
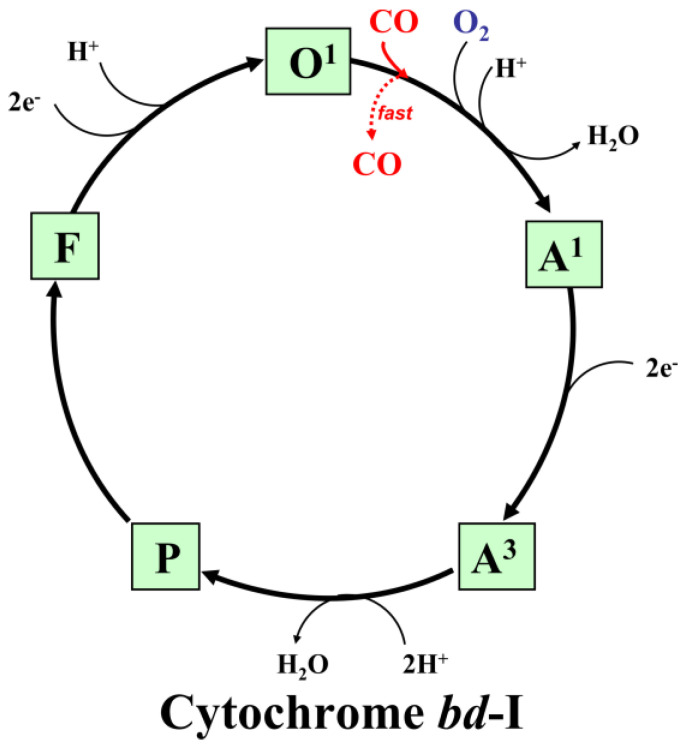
Proposed mechanism for the inhibitory effects of CO on the catalytic cycle of cytochrome *bd*-I. Proposed catalytic intermediates **O^1^** (*b*_558_^2+^ *b*_595_^3+^ *d*^3+^–OH), **A^1^** (*b*_558_^3+^ *b*_595_^3+^ *d*^2+^–O_2_), **A^3^** (*b*_558_^2+^ *b*_595_^2+^ *d*^2+^–O_2_), **P** (*b*_558_^2+^ *b*_595_^3+^ d*^4+^=O^2^ where d*^4+^=O^2^ is a ferryl porphyrin π-cation radical [66,67]), and **F** (*b*_558_^3+^ *b*_595_^3+^ *d*^4+^=O^2−^) are shown. CO reacts with the **O^1^** species, yielding the *d*^2+^–CO complex, as suggested for cytochrome *bd*-II. However, at variance with cytochrome *bd*-II, in the case of cytochrome *bd*-I, due to the unusually high *off*-rate [69], CO does not bind with high affinity to heme *d*^2+^ and is rapidly ejected from the enzyme. As a result, CO does not affect its catalytic activity much. Also, in cytochrome *bd*-I, heme *b*_595_^2+^ does not bind CO to any significant extent [92]. These features thus presumably make cytochrome *bd*-I relatively insensitive to CO.

The possible molecular mechanisms of the inhibition of the catalytic activity of the *E. coli* cytochromes *bo*_3_, *bd*-II, and *bd*-I by CO are shown in Figure 8, Figure 9 and Figure 10, respectively (for more detail, see the legends to the figures).

In this work, we have used mutant strains, and this may present limitations, as mutations can potentially influence other processes in the cell that might go unnoticed and remain unaccounted for. However, the data on the wild-type cells and membranes are consistent with those obtained with the mutant strains. This gives us reason to believe that our conclusions are correct. As we mentioned above, the addition of CO triggers short-term alterations in the *E. coli* transcriptome [86], but a complete picture of the impact of CO on bacterial bioenergetics is lacking. Further studies are needed to shed light on long-term adaptation effects and fully uncover the extent of CO resistance in vivo.

## 4. Materials and Methods

### 4.1. Materials, E. coli Mutant Strains and Growth Conditions

The CO and N_2_ gases were purchased from Linde (Danbury, CT, USA) and Air Liquide (Air Liquide Italia Spa, Milano, Italy), respectively. Other chemicals were purchased from Merck KGaA (Darmstadt, Germany). A stock solution of CO or N_2_ was prepared by equilibrating degassed water with the pure gas at 1 atm and room temperature, yielding 1 mM CO and 0.7 mM N_2_ in solution. *E. coli* respiratory mutant strains TBE025 (MG1655 Δ*cydB nuoB appB::kan*), TBE026 (MG1655 Δ*cydB nuoB cyoB::kan*), and TBE037 (MG1655 Δ*appB nuoB cyoB::kan*), which respectively express cytochrome *bo*_3_, cytochrome *bd*-II, or cytochrome *bd*-I as the sole terminal oxidase, were used [77]. *E. coli* cultures were grown in Luria–Bertani (LB) medium supplemented with 30 μg/mL kanamycin, at 37 °C and 200 rpm. In the case of growth studies, after inoculation at an OD_600_ of 0.15 ± 0.03, the cells were grown in 50 mL rubber-stoppered flasks in 16 mL of air-equilibrated LB at 37 °C for 60 min; when OD_600_ reached 0.4 ± 0.08, they were bubbled for 30 s with either CO (pure gas at 1 atm and room temperature) or N_2_ (pure gas at 1 atm and room temperature) as a control to yield a final concentration of both gases of ~20%. The gas percentage was assessed by taking into account the different O_2_ concentrations, present in equal volumes of LB equilibrated with air or flushed with CO/N_2_ for 30 s, and calculated according to the following equation: Gas % = ([O_2_] _air equilibrated_ − [O_2_] _gas flushed_)/[O_2_] _air equilibrated_ × 100. [O_2_] _air equilibrated_ and [O_2_] _gas flushed_ are the O_2_ concentration values measured in the degassed chamber of a high-resolution respirometer (Oxygraph-2k, Oroboros Instruments GmbH, Innsbruck, Austria) of non-fluxed and fluxed *E. coli* cultures with CO or N_2_, respectively. The growth of the *E. coli* cultures was then monitored via a standard method using optical density measurements in an Eppendorf BioSpectrometer basic at 600 nm every 30 min. When the OD_600_ was above 1, cultures were diluted before reading.

### 4.2. Investigation of the Effect of CO on Respiration of Wild-Type E. coli Cells

To assess the effect of CO on the respiration of wild-type *E. coli* cells (strain MG1655), we investigated aerobic cultures in which a change in oxidase expression from cytochrome *bo*_3_ to the *bd*-type cytochromes is expected to occur during cell growth, following a progressive reduction in the availability of O_2_ in the medium, taking into account the striking difference between the oxidases in sensitivity to sodium sulfide, according to Forte et al. [77]. When cells grown in Luria–Bertani (LB) medium are assayed in an early growth phase of the culture (OD_600_ < 0.8), most of the respiration (50–70%) is sensitive to 50 μM sodium sulfide, indicating a prevalent expression of cytochrome *bo*_3_. In contrast, in a late growth phase of the culture (OD_600_ > 2.5), when oxygen is limiting, sodium sulfide causes only marginal effects on respiration, indicating a prevalent expression of the *bd*-type cytochromes.

### 4.3. Isolation of Membranes from E. coli Respiratory Mutants

To isolate membranes from *E. coli* respiratory mutants expressing only one terminal quinol oxidase (cytochrome *bd*-I or cytochrome *bd*-II or cytochrome *bo*_3_), cell cultures were grown in LB medium until an OD_600_ of about 2 was reached. The cells were then pelleted by centrifugation at 10,000 rpm for 10 min and washed twice in 20 mM TRIS pH 8.3 containing 0.5 mM EDTA and 5 mM MgSO_4_. The cells were resuspended in the same buffer containing 1 mg/mL lysozyme and incubated for two hours on ice. A spatula tip of DNAse and RNase was then added, and the cells were lysed by sonication. Cell debris was removed by centrifugation at 15,000 rpm for 10 min. Membrane fractions were collected and stored at −80 °C. The protein content was determined using the Bradford method, using the Bradford reagent (Sigma) with bovine serum albumin as standard.

### 4.4. Oxygraphic Measurements

Oxygraphic measurements were performed at 25 °C in 50 mM K/phosphate pH 7.0, using a high-resolution respirometer (Oxygraph-2k, Oroboros Instruments GmbH, Innsbruck, Austria) equipped with two 1.5-mL chambers. The O_2_ consumption of *E. coli* cells was followed with endogenous reductants. In the case of isolated membranes, O_2_ consumption was measured in the presence of 2.5 mM dithiothreitol (DTT) and 2.5 μM 2,3-dimethoxy-5-methyl-6-(3-methyl-2-butenyl)-1,4-benzoquinone (Q_1_), an artificial reducing couple specific for quinol oxidases. The catalytic O_2_-consuming activity was obtained by subtracting the non-enzymatic O_2_ consumption (Q_1_/DTT autoxidation in the absence of the membranes) from the O_2_ consumption rate measured after the addition of membranes.

### 4.5. Spectroscopic Measurements

UV–visible absorption spectra were recorded to estimate the amount of each terminal oxidase present in cell suspensions. For this purpose, an Agilent Cary 60 UV-Vis or a Varian Cary 300 Bio UV-Visible spectrophotometer was used. The amount of oxidase present in each strain was estimated from the dithionite-reduced-*minus*-ferricyanyde-oxidized difference absorption spectrum of sonicated cells using Δ*ε*_561–580_ of 21 mM^−1^ cm^−1^ (*bd*-I and *bd*-II) [92] and 16.3 mM^−1^ cm^−1^ (*bo*_3_) [88].

### 4.6. Data Analysis

Data analysis was carried out using software packages Origin v7.0 (OriginLab Corporation, Northampton, MA, USA) and GIM (Scientific Graphic Interactive Management System developed by A.L. Drachev in Lomonosov Moscow State University). The percentage inhibition of O_2_ consumption of cell suspensions (*i*%) was calculated using the equation *i*% = ((V_0_ − V_0,i_)/V_0_)·100, where V_0_ and V_0,i_ are the initial rates in the absence and in the presence of the inhibitor (CO), respectively. The apparent *IC*_50_ values of cytochromes *bd*-II and *bo*_3_ for CO were estimated by plotting *i*% as a function of CO concentration added ([CO]_0_). The data were fitted to the standard hyperbolic equation *i*% = *i*_max_%·[CO]_0_/(*IC*_50_ + [CO]_0_) using a built-in approximation function (‘Hyperbola function’) in ‘Advanced Fitting tool’ in the Origin program. The *i*_max_% parameter is a theoretical maximum percent inhibition. The inhibition constants (*K*_i_) of cytochromes *bd*-II and *bo*_3_ for CO were estimated by plotting, as a function of [O_2_]/*K*_m_(O_2_), the *IC*_50_ values measured at different O_2_ concentrations and fitting the data to the equation *IC*_50_ = (*K*_i_·[O_2_]/*K*_m_(O_2_)) + *K*_i_ [91], assuming O_2_ competitive inhibition. The apparent *K*_m_(O_2_) values 2 µM (for cytochrome *bd*-II) and 6 µM (for cytochrome *bo*_3_) were taken from [90] and [70], respectively.

Statistical analyses were performed using an unpaired t-test for comparisons between two strains or conditions.

## 5. Conclusions

In this study, we show that of the two *E. coli bd*-type oxidases, only *bd*-I promotes growth in the presence of toxic concentrations of CO, giving the bacterium resistance to the gas. Our results are relevant for clarifying the discrepancies present in the literature on the effect of CO on the O_2_ consumption of *E. coli* oxidases and expanding the knowledge of cytochromes *bd*, a protein family of increasing interest due to their unique functional and structural features and their importance to pathogens. These findings are also important in the field of microbial physiology as they contribute to a better understanding of how different terminal complexes participate in the respiratory chain under various growth conditions. Moreover, they may provide a basis for biotechnological applications in which an increased bacterial resistance to CO is needed [100]. Finally, we believe that these findings could have a biomedical significance. These data should be taken into account when next-generation antimicrobials, whose mechanism of action is based on the release of CO that blocks the aerobic respiration and growth of pathogenic bacteria, are studied for potential use in medicine. Therapeutic CO delivery in humans and animals by such CO-releasing molecules would most likely be less efficient if the target pathogen possesses a terminal oxidase similar to the *E. coli bd*-I enzyme.

## Data Availability

The data presented in this study are contained in the article and the Supplementary Materials.

## Supplementary Materials

The supporting information can be downloaded at: https://www.mdpi.com/article/10.3390/ijms25021277/s1.

## References

[1] Ingi T., Ronnett G.V. (1995). Direct demonstration of a physiological role for carbon monoxide in olfactory receptor neurons. J. Neurosci..

[2] Friebe A., Schultz G., Koesling D. (1996). Sensitizing soluble guanylyl cyclase to become a highly CO-sensitive enzyme. Embo J..

[3] Siow R.C., Sato H., Mann G.E. (1999). Heme oxygenase-carbon monoxide signalling pathway in atherosclerosis: Anti-atherogenic actions of bilirubin and carbon monoxide?. Cardiovasc. Res..

[4] Boehning D., Snyder S.H. (2002). Circadian rhythms. Carbon monoxide and clocks. Science.

[5] Morse D., Sethi J., Choi A.M. (2002). Carbon monoxide-dependent signaling. Crit. Care Med..

[6] Ryter S.W., Otterbein L.E., Morse D., Choi A.M. (2002). Heme oxygenase/carbon monoxide signaling pathways: Regulation and functional significance. Mol. Cell Biochem..

[7] Bilban M., Haschemi A., Wegiel B., Chin B.Y., Wagner O., Otterbein L.E. (2008). Heme oxygenase and carbon monoxide initiate homeostatic signaling. J. Mol. Med. (Berl.).

[8] Piantadosi C.A. (2008). Carbon monoxide, reactive oxygen signaling, and oxidative stress. Free Radic. Biol. Med..

[9] Olson K.R., Donald J.A. (2009). Nervous control of circulation-the role of gasotransmitters, NO, CO, and H_2_S. Acta Histochem..

[10] Wang X.M., Kim H.P., Nakahira K., Ryter S.W., Choi A.M. (2009). The heme oxygenase-1/carbon monoxide pathway suppresses TLR4 signaling by regulating the interaction of TLR4 with caveolin-1. J. Immunol..

[11] Farrugia G., Szurszewski J.H. (2014). Carbon monoxide, hydrogen sulfide, and nitric oxide as signaling molecules in the gastrointestinal tract. Gastroenterology.

[12] Choi Y.K., Maki T., Mandeville E.T., Koh S.H., Hayakawa K., Arai K., Kim Y.M., Whalen M.J., Xing C., Wang X. (2016). Dual effects of carbon monoxide on pericytes and neurogenesis in traumatic brain injury. Nat. Med..

[13] Wood H. (2016). Traumatic brain injury: Carbon monoxide-a potential therapy for traumatic brain injury?. Nat. Rev. Neurol..

[14] Kim H.J., Joe Y., Kim S.K., Park S.U., Park J., Chen Y., Kim J., Ryu J., Cho G.J., Surh Y.J. (2017). Carbon monoxide protects against hepatic steatosis in mice by inducing sestrin-2 via the PERK-eIF2alpha-ATF4 pathway. Free Radic. Biol. Med..

[15] Klemz R., Reischl S., Wallach T., Witte N., Jurchott K., Klemz S., Lang V., Lorenzen S., Knauer M., Heidenreich S. (2017). Reciprocal regulation of carbon monoxide metabolism and the circadian clock. Nat. Struct. Mol. Biol..

[16] Correa-Costa M., Gallo D., Csizmadia E., Gomperts E., Lieberum J.L., Hauser C.J., Ji X., Wang B., Camara N.O.S., Robson S.C. (2018). Carbon monoxide protects the kidney through the central circadian clock and CD39. Proc. Natl. Acad. Sci. USA.

[17] Joe Y., Kim S., Kim H.J., Park J., Chen Y., Park H.J., Jekal S.J., Ryter S.W., Kim U.H., Chung H.T. (2018). FGF21 induced by carbon monoxide mediates metabolic homeostasis via the PERK/ATF4 pathway. Faseb J..

[18] Minegishi S., Sagami I., Negi S., Kano K., Kitagishi H. (2018). Circadian clock disruption by selective removal of endogenous carbon monoxide. Sci. Rep..

[19] Chen Y., Joe Y., Park J., Song H.C., Kim U.H., Chung H.T. (2019). Carbon monoxide induces the assembly of stress granule through the integrated stress response. Biochem. Biophys. Res. Commun..

[20] Rahman F.U., Park D.R., Joe Y., Jang K.Y., Chung H.T., Kim U.H. (2019). Critical Roles of Carbon Monoxide and Nitric Oxide in Ca(2+) Signaling for Insulin Secretion in Pancreatic Islets. Antioxid. Redox Signal..

[21] Stucki D., Steinhausen J., Westhoff P., Krahl H., Brilhaus D., Massenberg A., Weber A.P.M., Reichert A.S., Brenneisen P., Stahl W. (2020). Endogenous Carbon Monoxide Signaling Modulates Mitochondrial Function and Intracellular Glucose Utilization: Impact of the Heme Oxygenase Substrate Hemin. Antioxidants.

[22] Park J., Zeng J.S., Sahasrabudhe A., Jin K., Fink Y., Manthiram K., Anikeeva P. (2021). Electrochemical Modulation of Carbon Monoxide-Mediated Cell Signaling. Angew. Chem. Int. Ed. Engl..

[23] Yuan Z., De La Cruz L.K., Yang X., Wang B. (2022). Carbon monoxide signaling: Examining its engagement with various molecular targets in the context of binding affinity, concentration, and biologic response. Pharmacol. Rev..

[24] Hopper C.P., De La Cruz L.K., Lyles K.V., Wareham L.K., Gilbert J.A., Eichenbaum Z., Magierowski M., Poole R.K., Wollborn J., Wang B. (2020). Role of carbon monoxide in host-gut microbiome communication. Chem. Rev..

[25] Dent M.R., Weaver B.R., Roberts M.G., Burstyn J.N. (2023). Carbon monoxide-sensing transcription factors: Regulators of microbial carbon monoxide oxidation pathway gene expression. J. Bacteriol..

[26] Tavares A.F., Parente M.R., Justino M.C., Oleastro M., Nobre L.S., Saraiva L.M. (2013). The bactericidal activity of carbon monoxide-releasing molecules against *Helicobacter pylori*. PLoS ONE.

[27] Desmard M., Davidge K.S., Bouvet O., Morin D., Roux D., Foresti R., Ricard J.D., Denamur E., Poole R.K., Montravers P. (2009). A carbon monoxide-releasing molecule (CORM-3) exerts bactericidal activity against *Pseudomonas aeruginosa* and improves survival in an animal model of bacteraemia. Faseb J..

[28] Davidge K.S., Sanguinetti G., Yee C.H., Cox A.G., McLeod C.W., Monk C.E., Mann B.E., Motterlini R., Poole R.K. (2009). Carbon monoxide-releasing antibacterial molecules target respiration and global transcriptional regulators. J. Biol. Chem..

[29] Wareham L.K., McLean S., Begg R., Rana N., Ali S., Kendall J.J., Sanguinetti G., Mann B.E., Poole R.K. (2018). The broad-spectrum antimicrobial potential of [Mn(CO)_4_(S_2_CNMe(CH_2_CO_2_H))], a water-soluble CO-releasing molecule (CORM-401): Intracellular accumulation, transcriptomic and statistical analyses, and membrane polarization. Antioxid. Redox. Signal..

[30] Southam H.M., Smith T.W., Lyon R.L., Liao C., Trevitt C.R., Middlemiss L.A., Cox F.L., Chapman J.A., El-Khamisy S.F., Hippler M. (2018). A thiol-reactive Ru(II) ion, not CO release, underlies the potent antimicrobial and cytotoxic properties of CO-releasing molecule-3. Redox Biol..

[31] Sousa F.L., Alves R.J., Ribeiro M.A., Pereira-Leal J.B., Teixeira M., Pereira M.M. (2012). The superfamily of heme-copper oxygen reductases: Types and evolutionary considerations. Biochim. Biophys. Acta.

[32] Murali R., Gennis R.B., Hemp J. (2021). Evolution of the cytochrome *bd* oxygen reductase superfamily and the function of CydAA’ in Archaea. ISME J..

[33] Siletsky S.A. (2023). Investigation of the Mechanism of Membrane Potential Generation by Heme-Copper Respiratory Oxidases in a Real Time Mode. Biochemistry.

[34] Safari C., Ghosh S., Andersson R., Johannesson J., Bath P., Uwangue O., Dahl P., Zoric D., Sandelin E., Vallejos A. (2023). Time-resolved serial crystallography to track the dynamics of carbon monoxide in the active site of cytochrome c oxidase. Sci. Adv..

[35] Moe A., Dimogkioka A.R., Rapaport D., Ojemyr L.N., Brzezinski P. (2023). Structure and function of the S. pombe III-IV-cyt c supercomplex. Proc. Natl. Acad. Sci. USA.

[36] Khalfaoui-Hassani B., Blaby-Haas C.E., Verissimo A., Daldal F. (2023). The Escherichia coli MFS-type transporter genes yhjE, ydiM, and yfcJ are required to produce an active bo3 quinol oxidase. PLoS ONE.

[37] Shimada A., Etoh Y., Kitoh-Fujisawa R., Sasaki A., Shinzawa-Itoh K., Hiromoto T., Yamashita E., Muramoto K., Tsukihara T., Yoshikawa S. (2020). X-ray structures of catalytic intermediates of cytochrome *c* oxidase provide insights into its O_2_ activation and unidirectional proton-pump mechanisms. J. Biol. Chem..

[38] Shimada A., Hara F., Shinzawa-Itoh K., Kanehisa N., Yamashita E., Muramoto K., Tsukihara T., Yoshikawa S. (2021). Critical roles of the Cu_B_ site in efficient proton pumping as revealed by crystal structures of mammalian cytochrome *c* oxidase catalytic intermediates. J. Biol. Chem..

[39] Shimada A., Baba J., Nagao S., Shinzawa-Itoh K., Yamashita E., Muramoto K., Tsukihara T., Yoshikawa S. (2023). Crystallographic cyanide-probing for cytochrome c oxidase reveals structural bases suggesting that a putative proton transfer H-pathway pumps protons. J. Biol. Chem..

[40] Panda S., Phan H., Karlin K.D. (2023). Heme-copper and Heme O_2_-derived synthetic (bioinorganic) chemistry toward an understanding of cytochrome c oxidase dioxygen chemistry. J. Inorg. Biochem..

[41] Ishigami I., Sierra R.G., Su Z., Peck A., Wang C., Poitevin F., Lisova S., Hayes B., Moss F.R., Boutet S. (2023). Structural insights into functional properties of the oxidized form of cytochrome c oxidase. Nat. Commun..

[42] Verameyenka K.G., Naumouskaya V.A., Maximova N.P. (2023). Cytochrome c oxidase is one of the key enzymes providing the ability to produce phenazines in Pseudomonas chlororaphis subsp. aurantiaca. World J. Microbiol. Biotechnol..

[43] Guo Y., Karimullina E., Emde T., Otwinowski Z., Borek D., Savchenko A. (2023). Monomer and dimer structures of cytochrome *bo_3_* ubiquinol oxidase from *Escherichia coli*. Protein Sci..

[44] Li J., Han L., Vallese F., Ding Z., Choi S.K., Hong S., Luo Y., Liu B., Chan C.K., Tajkhorshid E. (2021). Cryo-EM structures of *Escherichia coli* cytochrome *bo_3_* reveal bound phospholipids and ubiquinone-8 in a dynamic substrate binding site. Proc. Natl. Acad. Sci. USA.

[45] Jose A., Schaefer A.W., Roveda A.C., Transue W.J., Choi S.K., Ding Z., Gennis R.B., Solomon E.I. (2021). The three-spin intermediate at the O-O cleavage and proton-pumping junction in heme-Cu oxidases. Science.

[46] Asseri A.H., Godoy-Hernandez A., Goojani H.G., Lill H., Sakamoto J., McMillan D.G.G., Bald D. (2021). Cardiolipin enhances the enzymatic activity of cytochrome *bd* and cytochrome *bo*_3_ solubilized in dodecyl-maltoside. Sci. Rep..

[47] Kaila V.R.I., Wikstrom M. (2021). Architecture of bacterial respiratory chains. Nat. Rev. Microbiol..

[48] Wikstrom M., Springett R. (2020). Thermodynamic efficiency, reversibility, and degree of coupling in energy conservation by the mitochondrial respiratory chain. Commun. Biol..

[49] Di Trani J.M., Gheorghita A.A., Turner M., Brzezinski P., Adelroth P., Vahidi S., Howell P.L., Rubinstein J.L. (2023). Structure of the *bc*_1_-*cbb*_3_ respiratory supercomplex from *Pseudomonas aeruginosa*. Proc. Natl. Acad. Sci. USA.

[50] Zhang L., Dong T., Yang J., Hao S., Sun Z., Peng Y. (2023). Anammox Coupled with Photocatalyst for Enhanced Nitrogen Removal and the Activated Aerobic Respiration of Anammox Bacteria Based on *cbb*_3_-Type Cytochrome *c* Oxidase. Environ. Sci. Technol..

[51] Yang X., Liu S., Yin Z., Chen M., Song J., Li P., Yang L. (2023). New insights into the proton pumping mechanism of *ba*_3_ cytochrome *c* oxidase: The functions of key residues and water. Phys. Chem. Chem. Phys..

[52] Noodleman L., Gotz A.W., Han Du W.G., Hunsicker-Wang L. (2023). Reaction pathways, proton transfer, and proton pumping in *ba*_3_ class cytochrome *c* oxidase: Perspectives from DFT quantum chemistry and molecular dynamics. Front. Chem..

[53] Wikstrom M., Pecorilla C., Sharma V. (2023). The mitochondrial respiratory chain. Enzymes.

[54] Wikstrom M., Gennis R.B., Rich P.R. (2023). Structures of the intermediates in the catalytic cycle of mitochondrial cytochrome *c* oxidase. Biochim. Biophys. Acta. Bioenerg..

[55] Poole R.K., Cook G.M. (2000). Redundancy of aerobic respiratory chains in bacteria? Routes, reasons and regulation. Adv. Microb. Physiol..

[56] Melo A.M., Teixeira M. (2016). Supramolecular organization of bacterial aerobic respiratory chains: From cells and back. Biochim. Biophys. Acta.

[57] Melin F., Sabuncu S., Choi S.K., Leprince A., Gennis R.B., Hellwig P. (2018). Role of the tightly bound quinone for the oxygen reaction of cytochrome *bo_3_* oxidase from *Escherichia coli*. FEBS Lett..

[58] Borisov V.B., Forte E. (2021). Impact of hydrogen sulfide on mitochondrial and bacterial bioenergetics. Int. J. Mol. Sci..

[59] Borisov V.B., Siletsky S.A., Paiardini A., Hoogewijs D., Forte E., Giuffre A., Poole R.K. (2021). Bacterial oxidases of the cytochrome *bd* family: Redox enzymes of unique structure, function and utility as drug targets. Antioxid. Redox Signal..

[60] Rolfe M.D., Ter Beek A., Graham A.I., Trotter E.W., Asif H.M., Sanguinetti G., de Mattos J.T., Poole R.K., Green J. (2011). Transcript profiling and inference of *Escherichia coli* K-12 ArcA activity across the range of physiologically relevant oxygen concentrations. J. Biol. Chem..

[61] Alexeeva S., Hellingwerf K., Teixeira de Mattos M.J. (2002). Quantitative assessment of oxygen availability: Perceived aerobiosis and its effect on flux distribution in the respiratory chain of *Escherichia coli*. J. Bacteriol..

[62] Trotter E.W., Rolfe M.D., Hounslow A.M., Craven C.J., Williamson M.P., Sanguinetti G., Poole R.K., Green J. (2011). Reprogramming of *Escherichia coli* K-12 metabolism during the initial phase of transition from an anaerobic to a micro-aerobic environment. PLoS ONE.

[63] Abramson J., Riistama S., Larsson G., Jasaitis A., Svensson-Ek M., Laakkonen L., Puustinen A., Iwata S., Wikstrom M. (2000). The structure of the ubiquinol oxidase from *Escherichia coli* and its ubiquinone binding site. Nat. Struct. Biol..

[64] Safarian S., Hahn A., Mills D.J., Radloff M., Eisinger M.L., Nikolaev A., Meier-Credo J., Melin F., Miyoshi H., Gennis R.B. (2019). Active site rearrangement and structural divergence in prokaryotic respiratory oxidases. Science.

[65] Thesseling A., Rasmussen T., Burschel S., Wohlwend D., Kagi J., Muller R., Bottcher B., Friedrich T. (2019). Homologous *bd* oxidases share the same architecture but differ in mechanism. Nat. Commun..

[66] Grauel A., Kagi J., Rasmussen T., Makarchuk I., Oppermann S., Moumbock A.F.A., Wohlwend D., Muller R., Melin F., Gunther S. (2021). Structure of *Escherichia coli* cytochrome *bd*-II type oxidase with bound aurachin D. Nat. Commun..

[67] Grund T.N., Radloff M., Wu D., Goojani H.G., Witte L.F., Josting W., Buschmann S., Muller H., Elamri I., Welsch S. (2021). Mechanistic and structural diversity between cytochrome *bd* isoforms of *Escherichia coli*. Proc. Natl. Acad. Sci. USA.

[68] Samukaite Bubniene U., Zukauskas S., Ratautaite V., Vilkiene M., Mockeviciene I., Liustrovaite V., Drobysh M., Lisauskas A., Ramanavicius S., Ramanavicius A. (2022). Assessment of cytochrome c and chlorophyll a as natural redox mediators for enzymatic biofuel cells powered by glucose. Energies.

[69] Borisov V.B., Forte E., Sarti P., Brunori M., Konstantinov A.A., Giuffre A. (2007). Redox control of fast ligand dissociation from *Escherichia coli* cytochrome *bd*. Biochem. Biophys. Res. Commun..

[70] Mason M.G., Shepherd M., Nicholls P., Dobbin P.S., Dodsworth K.S., Poole R.K., Cooper C.E. (2009). Cytochrome *bd* confers nitric oxide resistance to *Escherichia coli*. Nat. Chem. Biol..

[71] Shepherd M., Achard M.E., Idris A., Totsika M., Phan M.D., Peters K.M., Sarkar S., Ribeiro C.A., Holyoake L.V., Ladakis D. (2016). The cytochrome *bd*-I respiratory oxidase augments survival of multidrug-resistant *Escherichia coli* during infection. Sci. Rep..

[72] Borisov V.B., Forte E., Siletsky S.A., Sarti P., Giuffre A. (2015). Cytochrome *bd* from *Escherichia coli* catalyzes peroxynitrite decomposition. Biochim. Biophys. Acta.

[73] Forte E., Siletsky S.A., Borisov V.B. (2021). In *Escherichia coli* ammonia inhibits cytochrome *bo*_3_ but activates cytochrome *bd*-I. Antioxidants.

[74] Sarti P., Giuffre A., Forte E., Mastronicola D., Barone M.C., Brunori M. (2000). Nitric oxide and cytochrome *c* oxidase: Mechanisms of inhibition and NO degradation. Biochem. Biophys. Res. Commun..

[75] Borisov V.B., Forte E., Sarti P., Brunori M., Konstantinov A.A., Giuffre A. (2006). Nitric oxide reacts with the ferryl-oxo catalytic intermediate of the Cu_B_-lacking cytochrome *bd* terminal oxidase. FEBS Lett..

[76] Borisov V.B., Forte E. (2022). Bioenergetics and reactive nitrogen species in bacteria. Int. J. Mol. Sci..

[77] Forte E., Borisov V.B., Falabella M., Colaco H.G., Tinajero-Trejo M., Poole R.K., Vicente J.B., Sarti P., Giuffre A. (2016). The terminal oxidase cytochrome *bd* promotes sulfide-resistant bacterial respiration and growth. Sci. Rep..

[78] Borisov V.B., Forte E., Davletshin A., Mastronicola D., Sarti P., Giuffre A. (2013). Cytochrome *bd* oxidase from *Escherichia coli* displays high catalase activity: An additional defense against oxidative stress. FEBS Lett..

[79] Al-Attar S., Yu Y., Pinkse M., Hoeser J., Friedrich T., Bald D., de Vries S. (2016). Cytochrome *bd* displays significant quinol peroxidase activity. Sci. Rep..

[80] Forte E., Nastasi M.R., Borisov V.B. (2022). Preparations of terminal oxidase cytochrome *bd*-II isolated from *Escherichia coli* reveal significant hydrogen peroxide scavenging activity. Biochemistry.

[81] Korshunov S., Imlay K.R., Imlay J.A. (2016). The cytochrome *bd* oxidase of *Escherichia coli* prevents respiratory inhibition by endogenous and exogenous hydrogen sulfide. Mol. Microbiol..

[82] Harikishore A., Mathiyazakan V., Pethe K., Gruber G. (2023). Novel targets and inhibitors of the *Mycobacterium tuberculosis* cytochrome *bd* oxidase to foster anti-tuberculosis drug discovery. Expert Opin. Drug Discov..

[83] Bayly K., Cordero P.R.F., Kropp A., Huang C., Schittenhelm R.B., Grinter R., Greening C. (2021). Mycobacteria tolerate carbon monoxide by remodeling their respiratory chain. mSystems.

[84] Scharn C.R., Collins A.C., Nair V.R., Stamm C.E., Marciano D.K., Graviss E.A., Shiloh M.U. (2016). Heme oxygenase-1 regulates inflammation and mycobacterial survival in human macrophages during *Mycobacterium tuberculosis* infection. J. Immunol..

[85] Wegiel B., Larsen R., Gallo D., Chin B.Y., Harris C., Mannam P., Kaczmarek E., Lee P.J., Zuckerbraun B.S., Flavell R. (2014). Macrophages sense and kill bacteria through carbon monoxide-dependent inflammasome activation. J. Clin. Invest..

[86] Wareham L.K., Begg R., Jesse H.E., Van Beilen J.W., Ali S., Svistunenko D., McLean S., Hellingwerf K.J., Sanguinetti G., Poole R.K. (2016). Carbon monoxide gas is not inert, but global, in its consequences for bacterial gene expression, iron acquisition, and antibiotic resistance. Antioxid. Redox. Signal..

[87] Forte E., Borisov V.B., Siletsky S.A., Petrosino M., Giuffre A. (2019). In the respiratory chain of *Escherichia coli* cytochromes *bd*-I and *bd*-II are more sensitive to carbon monoxide inhibition than cytochrome *bo*_3_. Biochim. Biophys. Acta Bioenerg..

[88] Nastasi M.R., Borisov V.B., Forte E. (2023). The terminal oxidase cytochrome *bd*-I confers carbon monoxide resistance to *Escherichia coli* cells. J. Inorg. Biochem..

[89] Kalia N.P., Singh S., Hards K., Cheung C.Y., Sviriaeva E., Banaei-Esfahani A., Aebersold R., Berney M., Cook G.M., Pethe K.M. (2023). *Tuberculosis* relies on trace oxygen to maintain energy homeostasis and survive in hypoxic environments. Cell Rep..

[90] Bekker M., de Vries S., Ter Beek A., Hellingwerf K.J., de Mattos M.J. (2009). Respiration of *Escherichia coli* can be fully uncoupled via the nonelectrogenic terminal cytochrome *bd*-II oxidase. J. Bacteriol..

[91] Cheng Y., Prusoff W.H. (1973). Relationship between the inhibition constant (*K_I_*) and the concentration of inhibitor which causes 50 per cent inhibition (*I*_50_) of an enzymatic reaction. Biochem. Pharmacol..

[92] Borisov V., Arutyunyan A.M., Osborne J.P., Gennis R.B., Konstantinov A.A. (1999). Magnetic circular dichroism used to examine the interaction of *Escherichia coli* cytochrome *bd* with ligands. Biochemistry.

[93] Puustinen A., Wikstrom M. (1991). The heme groups of cytochrome *o* from *Escherichia coli*. Proc. Natl. Acad. Sci. USA.

[94] Corradi V., Sejdiu B.I., Mesa-Galloso H., Abdizadeh H., Noskov S.Y., Marrink S.J., Tieleman D.P. (2019). Emerging diversity in lipid-protein interactions. Chem. Rev..

[95] Cournia Z., Allen T.W., Andricioaei I., Antonny B., Baum D., Brannigan G., Buchete N.V., Deckman J.T., Delemotte L., Del Val C. (2015). Membrane protein structure, function, and dynamics: A perspective from experiments and theory. J. Membr. Biol..

[96] Borisov V.B. (2020). Effect of membrane environment on ligand-binding properties of the terminal oxidase cytochrome *bd*-I from *Escherichia coli*. Biochemistry.

[97] Andreev I.M., Konstantinov A.A. (1983). Reaction of oxidized cytochrome oxidase with cyanide. Effects of pH, cytochrome *c* and membrane environment. Bioorg. Khim. (in Russian).

[98] Tsai A.L., Berka V., Martin E., Olson J.S. (2012). A "sliding scale rule" for selectivity among NO, CO, and O_2_ by heme protein sensors. Biochemistry.

[99] Bartlett G.J., Newberry R.W., VanVeller B., Raines R.T., Woolfson D.N. (2013). Interplay of hydrogen bonds and n-->pi* interactions in proteins. J. Am. Chem. Soc..

[100] Wickham-Smith C., Malys N., Winzer K. (2023). Improving carbon monoxide tolerance of *Cupriavidus necator* H16 through adaptive laboratory evolution. Front. Bioeng. Biotechnol..

[101] Proshlyakov D.A., Pressler M.A., DeMaso C., Leykam J.F., DeWitt D.L., Babcock G.T. (2000). Oxygen activation and reduction in respiration: Involvement of redox-active tyrosine 244. Science.

[102] Paulus A., Rossius S.G., Dijk M., de Vries S. (2012). Oxoferryl-porphyrin radical catalytic intermediate in cytochrome *bd* oxidases protects cells from formation of reactive oxygen species. J. Biol. Chem..

[103] Belevich I., Borisov V.B., Verkhovsky M.I. (2007). Discovery of the true peroxy intermediate in the catalytic cycle of terminal oxidases by real-time measurement. J. Biol. Chem..

