# Supplementary material for: Membrane-Bound Redox Enzyme Cytochrome bd-I Promotes Carbon Monoxide-Resistant Escherichia coli Growth and Respiration
Source: Int J Mol Sci. 2024 Jan 20;25(2):1277. doi: 10.3390/ijms25021277 (PMC10815991; doi:10.3390/ijms25021277)
Supplement: Supplementary file 1 [file ijms-25-01277-s001.zip › ijms-2750796-supplementary.pdf]

Supplementary Figures

Membrane-bound redox enzyme cytochrome *bd*-I promotes carbon monoxide-resistant *Escherichia coli* growth and respiration

Martina R. Nastasi [1], Vitaliy B. Borisov [2,*], Elena Forte [1,*]

[1] *Department of Biochemical Sciences, Sapienza University of Rome, 00185 Rome, Italy; martinaroberta.nastasi@uniroma1.it; Elena.Forte@uniroma1.it*
[2] *Belozersky Institute of Physico-Chemical Biology, Lomonosov Moscow State University, 119991 Moscow, Russia; bor@belozersky.msu.ru*

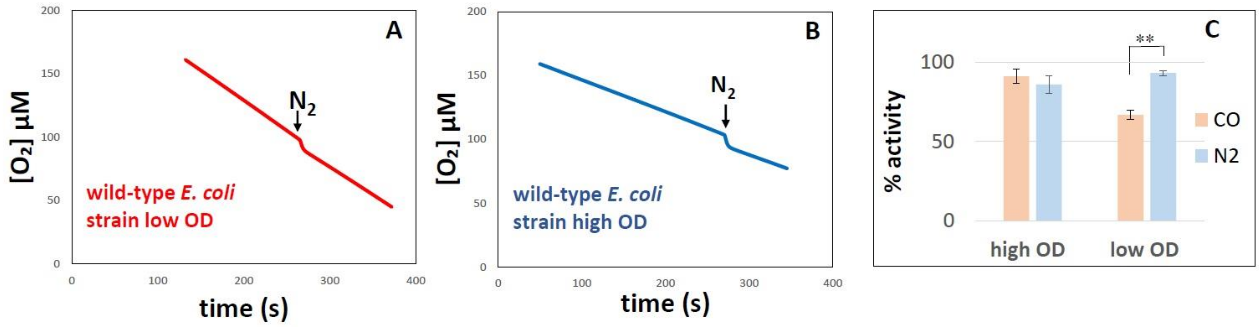

Supplementary Figure S1
Effect of N_2 on wild-type cell respiration. Traces of O_2 consumption of cell suspensions showing no effect of N_2 on aerobic respiration of the *E. coli* wild-type strain MG1655 measured with cells grown at low (**A**, red line) and at high (**B**, blue line) OD values. Low and high OD values indicate the prevalent expression of cytochrome *bo*3 and the *bd* oxidases, respectively. 96 µM N_2 was added at [O_2] = 100 µM. Additions: 1.5 ml of cells at 0.4 OD (red line), 0.25 ml of cells at 5 OD (blue line). (**C**): Percent activity after CO or N_2 addition to respiring wild-type cells. Values represent the mean (n = 3) ± standard deviations. Asterisks denote statistically significant differences between the effects of CO and N_2 in wild-type cells grown at low OD (**, p < 0,01; *t*-test).

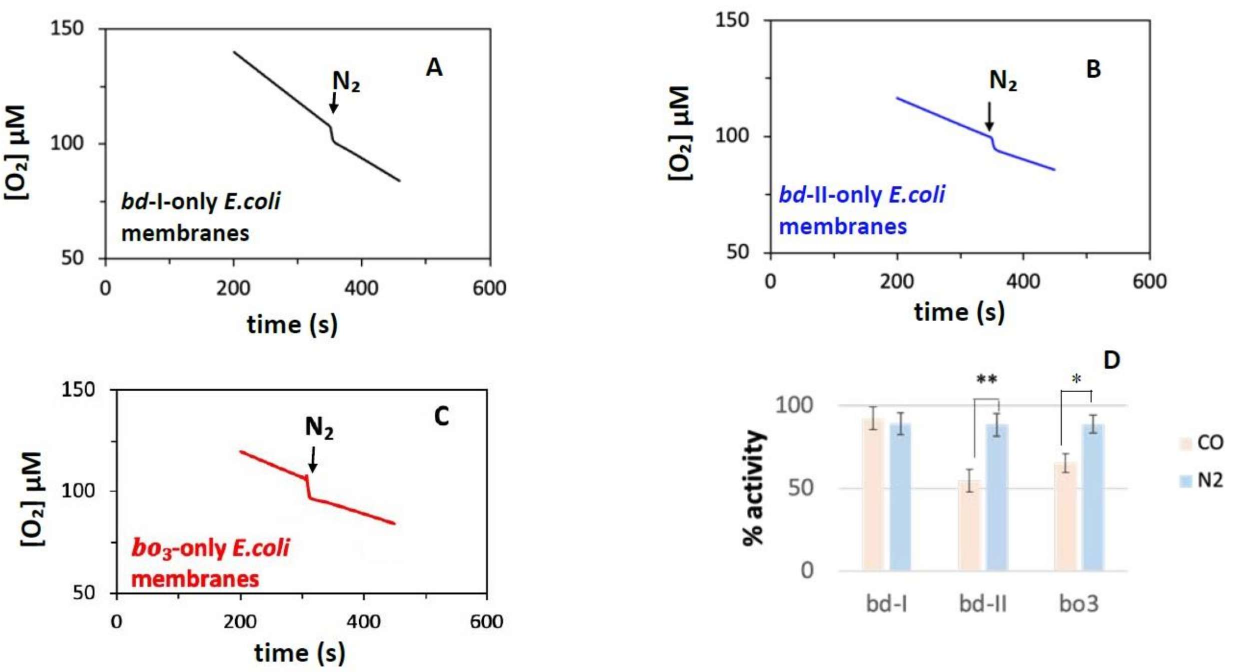

Supplementary Figure S2

Effect of N_2 on mutant strain respiration. Traces of O_2 consumption showing the effect of N_2 on aerobic respiration of isolated membranes from *E. coli* mutant strains expressing cytochrome *bd*-I (**A**), or cytochrome *bd*-II (**B**), or cytochrome *bo*$_3$ (**C**) as the sole terminal oxidase. 96 μM N_2 was added at [O_2] = 100 μM and had no effect on the respiratory activity of the membranes. Additions: 0.7 mg/ml of *bd*-I-containing isolated membranes (black line), 0.6 mg/ml of *bd*-II-containing isolated membranes (blue line), 0.7 mg/ml of *bo*$_3$-containing isolated membranes (red line). (**D**): Percent activity after CO or N_2 addition to respiring membranes. Values represent the mean (n = 3) ± standard deviations. Asterisks denote statistically significant differences between the effects of CO and N_2 in *bd*-II and *bo*$_3$ respiring isolated membranes (*, p < 0,05; **, p < 0,01; t-test).
